# Benchmarking Time-Series Artificial Intelligence Architectures for Wearable Sensor-Based Fall Prediction: A Synthetic Data Simulation Framework

**DOI:** 10.3390/s26113326

**Published:** 2026-05-24

**Authors:** Edward R. Sykes, Mohammad Maghsoudimehrabani, Abdulrahman Al-Shanoon

**Affiliations:** School of Computer Science, University of Guelph, Guelph, ON N1G 2W1, Canada; mmaghsou@uoguelph.ca (M.M.); alshanoa@uoguelph.ca (A.A.-S.)

**Keywords:** fall prediction, wearable sensors, multi-sensor fusion, time-series classification, synthetic data simulation, temporal deep learning, early warning, older adults

## Abstract

Falls among older adults are a major cause of injury and loss of independence, yet most existing systems detect falls only after onset or provide very limited warning time. This study presents a synthetic benchmarking framework for early fall-risk prediction using multimodal wearable-inspired time-series data and compares classical and temporal machine learning architectures under a realistic evaluation protocol. A synthetic dataset of 1000 sequences was generated to emulate normal activity, slip events, and pre-fall instability using biomechanical, physiological, and contextual variables. Eight baseline models and two augmented temporal variants were trained and evaluated using subject-wise splits to reduce leakage. Performance differed substantially by model family and evaluation protocol. Classical baselines achieved the strongest overall macro-F1 scores, whereas temporal models showed more modest discrimination. Under a fixed alerting rule, operational early-warning behavior varied considerably: some models failed to trigger alerts, while others achieved higher pre-fall trigger rates at the cost of increased false alarms. These findings show that apparent performance depends strongly on partitioning strategy, calibration, and alert design. The proposed framework provides a reproducible basis for benchmarking early-warning fall-risk models and supports future validation using real-world cohorts and deployment-oriented calibration strategies.

## 1. Introduction

Falls among older adults are a major cause of injury, hospitalization, and loss of independence [1,2]. As the global population ages, the need for accurate systems for fall prediction and fall detection becomes increasingly important. Existing fall detection systems are largely reactive, identifying falls only after they occur, which limits caregivers’ or clinicians’ ability to intervene before injury [3,4,5].

This study proposes a framework for an artificial intelligence (AI)-powered Fall Prediction System (FPS) that shifts the focus from post-fall detection to pre-fall risk prediction. The framework uses multi-sensor fusion to combine data from wearable devices with user-specific information, such as age and medical history, to support real-time risk estimation. The goal is to provide clinically meaningful warning time before a fall event and thereby improve opportunities for preventive intervention.

Despite continued progress in fall-related sensing and machine learning, several limitations still restrict practical deployment. Many existing datasets are based on younger, healthier participants performing scripted events, which limits their relevance to older adult populations [6,7,8]. In addition, many systems rely primarily on inertial data and do not incorporate richer physiological or contextual signals [9,10]. Another important limitation is lead time: many current approaches identify fall-related events only milliseconds to seconds before impact, which may be insufficient for meaningful intervention [11,12].

These limitations motivate the need for integrated, temporally aware fall prediction frameworks that combine multimodal sensor inputs with models capable of learning sequential patterns. Recent advances in recurrent neural networks (RNNs) and attention-based architectures have improved the modelling of sequential biomedical data [13,14], while wearable sensing technologies continue to expand the range of biomechanical and physiological variables available for monitoring [9,10]. In this work, we present a synthetic benchmarking framework for early fall-risk prediction using wearable-inspired multimodal time-series data. We compare classical machine learning baselines with temporal deep learning architectures under a subject-wise evaluation protocol and assess both classification performance and operational alerting behaviour.

From a sensor-systems perspective, the contribution of this work lies not only in model comparison but also in the design of a multimodal wearable sensing framework that integrates heterogeneous sensor inputs, temporal synchronization, and feature-level fusion, sensor-derived feature construction, and alert-oriented inference for fall-risk monitoring. The framework, therefore, emphasizes multimodal wearable sensing, feature-level fusion, and deployment-oriented alert generation for fall-risk monitoring.

## 2. Background

Falls among older adults remain a leading cause of injury, hospitalization, and loss of independence. While fall detection technologies have advanced, prediction remains limited by short lead times, constrained sensor data, and non-representative datasets. This section reviews five key areas influencing the development of effective fall prediction systems: dataset limitations, gait-based risk markers, biosignal relevance, sensor technologies, and current machine learning approaches.

### 2.1. Limitations of Current Fall Datasets

Existing fall datasets, including those reported by Auvinet et al., Kwolek and Kepski, and Feng et al., primarily rely on young, healthy participants performing scripted falls [6,7,8]. These scenarios lack ecological validity and fail to capture the subtle physiological and behavioural changes that may precede real falls in older adult populations. Furthermore, most existing datasets are limited to inertial sensor data and often exclude biosignals such as heart rate or blood pressure, which may carry useful predictive information.

### 2.2. Gait Analysis for Fall Risk Assessment

Gait characteristics such as step-time variability, stride length, and postural sway have long been used as indicators of fall risk [11,15]. Irregular gait patterns and increased double-support time may reflect compensatory mechanisms associated with impaired balance [16]. However, many falls are not preceded by obvious gait abnormalities and may instead arise from sudden physiological changes or environmental hazards. In addition, gait analysis often depends on specialized equipment, which limits its utility for continuous real-world monitoring.

### 2.3. Biosignals as Predictors of Fall Risk

Physiological signals such as heart rate, heart rate variability, respiratory rate, blood pressure, body temperature, and oxygen saturation offer complementary predictive value. Abnormal heart rate or respiratory rate, orthostatic hypotension, or oxygen saturation drops may indicate cardiovascular instability or acute physiological stress [17,18]. Although these signals are relevant to fall risk, they remain underused in many existing systems because of challenges associated with continuous acquisition and integration. The increasing availability of biosignal-capable wearable devices creates new opportunities for multimodal prediction frameworks.

### 2.4. Sensor Technologies for Fall Monitoring and Prediction

**Figure 1:**
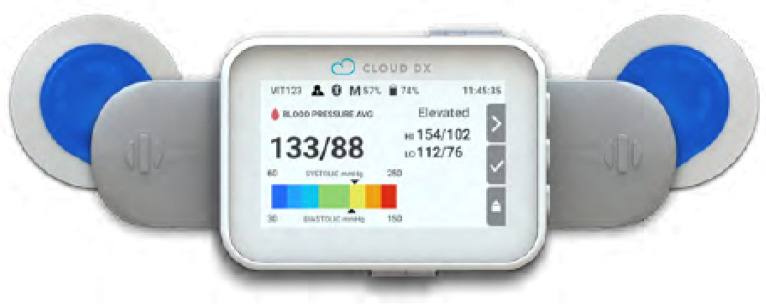
Cloud DX’s Vitaliti™ continuous patient monitoring wearable.

Modern wearable and ambient sensors enable continuous monitoring of both biomechanical and physiological parameters. Consumer devices such as smart watches and fitness trackers now include heart rate and oxygen saturation sensors, while more advanced devices support additional modalities such as electrocardiography and photoplethysmography. Advanced systems such as the Vitaliti™ wearable can monitor multiple vital signs, including heart rate, respiratory rate, blood pressure, temperature, oxygen saturation, and raw waveforms. Figure 1 illustrates the Vitaliti wearable. Other emerging platforms include smart textiles, pressure-sensing insoles, and footbed sensors that can track posture, balance, and centre-of-pressure dynamics in unobtrusive ways [9,10,19].

### 2.5. Current State of the Art in Fall Prediction Systems

Fall prediction has increasingly shifted from rule-based detection toward data-driven models. Traditional threshold-based approaches using accelerometer and gyroscope signals offer limited performance, especially for early prediction [15,20]. Recurrent neural network models such as Long Short-Term Memory (LSTM) and Gated Recurrent Unit (GRU) networks have shown promise for capturing temporal dependencies in sequential data [13]. More recently, transformer architectures have been explored for healthcare time-series modelling because of their ability to capture long-range dependencies using self-attention [14]. Despite these advances, many current systems still detect falls only milliseconds to seconds before impact, which restricts intervention potential [12].

### 2.6. Motivation

Collectively, these gaps in sensor diversity, ecological validity, and prediction horizon highlight the need for integrated, multimodal, and temporally aware fall prediction frameworks. This study addresses these challenges by combining wearable-inspired sensor variables with classical and temporal AI models in a synthetic benchmarking environment designed to support systematic comparison under realistic evaluation conditions.

## 3. Materials and Methods

### 3.1. Overview of the Proposed Framework

To address the limitations of existing fall prediction systems, we propose a user-centric AI-powered Fall Prediction System framework. The framework leverages multi-sensor fusion and time-series modelling to estimate fall risk in real time and generate actionable early warnings for caregivers and healthcare professionals. A central design feature is the integration of biomechanical, physiological, and contextual variables into a unified predictive pipeline.

**Figure 2:**
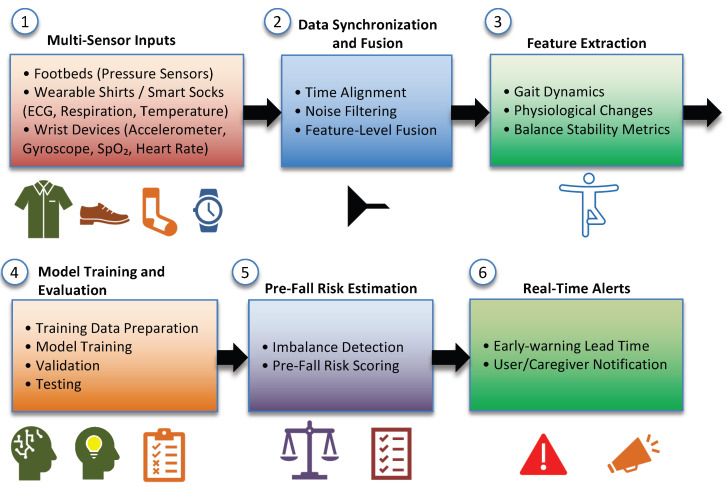
Proposed Fall Prediction System (FPS) framework. The system integrates multi-sensor inputs, data synchronization and fusion, feature extraction, model training and evaluation, pre-fall risk estimation, and real-time alert generation for caregiver notification.

Figure 2 presents the conceptual pipeline for the proposed framework, organized into six major steps: multi-sensor inputs, data synchronization and fusion, feature extraction, machine learning model training and testing, fall-risk prediction, and real-time alert generation.

### 3.2. Multi-Sensor Data Acquisition

The framework assumes data acquisition from multiple wearable platforms, including sensor-infused footbeds, smart socks, wearable garments, and wrist-worn devices. These sensing modalities are intended to capture complementary dimensions of fall risk: inertial sensors support motion and orientation tracking; pressure and footbed sensors support gait and balance assessment; and physiological sensors such as heart rate, respiration, and electrocardiogram signals support the detection of instability, fatigue, or acute physiological stress. These inputs may therefore include biomechanical and physiological variables such as heart rate, respiratory rate, electrocardiogram signals, accelerometer measurements, gyroscope measurements, and pressure-derived balance indicators. In addition to dynamic sensor streams, the framework incorporates static participant-level information such as age, weight, sex, medication profile, fall history, and relevant medical risk factors.

### 3.3. Synthetic Data Simulation

To support preliminary validation of the framework, we developed a synthetic dataset designed to emulate multimodal sensor data from older adults during routine activities, transient balance disturbances, and imminent falls. Each sequence represented a 60-s window sampled at 5 Hz, yielding 300 time steps per sequence. This sampling rate is consistent with many wearable sensor platforms and reflects a practical balance between signal fidelity and computational efficiency [10].

A total of 1000 sequences were generated and assigned to three behavioural categories: normal Activities of Daily Living (ADL) (70%), slip or misstep events (20%), and pre-fall imbalance states (10%). These class proportions were selected to approximate plausible monitoring conditions in which most recorded time is spent in routine activities, with relatively fewer transient disturbances and fewer high-risk pre-fall states [10,21,22,23].

### 3.4. Lead-Time Definition

To operationalize lead time in the synthetic benchmark, each pre-fall sequence was aligned to a simulated fall-onset anchor at the end of the 60-s window. Lead time was defined relative to this anchor and quantified using the operational alerting rule described below.

### 3.5. Operational Alerting Rule and Lead-Time Measurement

To evaluate whether classification outputs could support early warning, we simulated a simple streaming alerting rule over each test sequence. At each time point *t*, the trained model produced a predicted probability $p_{\mathrm{pre}\text{-}\mathrm{fall}}\left(t\right)$ for the pre-fall class. An alert was triggered when this probability exceeded a fixed threshold θ=0.70 for at least k=3 consecutive samples:

$$p_{\mathrm{pre}\text{-}\mathrm{fall}}\left(t\right)\geq \theta ,\;p_{\mathrm{pre}\text{-}\mathrm{fall}}\left(t-1\right)\geq \theta ,\;p_{\mathrm{pre}\text{-}\mathrm{fall}}\left(t-2\right)\geq \theta .$$

Because sequences were sampled at 5 Hz, this persistence requirement corresponds to approximately 0.6 s of sustained elevated predicted risk. The purpose of this rule was to reduce isolated probability spikes that could otherwise produce unstable alerts.

For each true pre-fall sequence, lead time was computed as the difference between the simulated fall-onset anchor at the end of the sequence window and the first alert time. If no alert was triggered before the anchor, the lead time was considered undefined for that sequence. For non-fall sequences (normal and slip), any alert was counted as a false alarm. We therefore report the pre-fall trigger rate, the non-fall false-alarm rate, and the median/interquartile lead times for triggered pre-fall sequences.

The threshold θ=0.70 and persistence parameter k=3 were selected as an illustrative operating point to demonstrate the sensitivity–false-alarm trade-off rather than as clinically optimized parameters. In practical deployment, thresholds would need to be calibrated for each model and care setting based on acceptable false-alarm burden, desired sensitivity, and intervention time requirements.

### 3.6. Empirical Grounding for Synthetic Data Generation

The synthetic generation process was informed by three categories of prior resources: video datasets with annotated fall phases, wearable and inertial measurement unit datasets capturing activities of daily living and falls, and balance or biosignal datasets containing centre-of-pressure and postural sway measures.

For behavioural grounding, we referenced the UR Fall Detection and Le2i datasets to characterize fall onset timing and phase progression [6,24]. For kinematic grounding, we aligned signal dynamics with established inertial datasets including MobiFall, MobiAct, and UP-Fall [25,26,27]. For physiological and balance-related grounding, we drew on publicly available resources indexed through PhysioNet, together with prior work on postural sway and centre-of-pressure markers [21,28,29].

This empirical grounding informed the expected value ranges, temporal evolution, and variability patterns for the simulated features. The intent was not to replicate any single source exactly, but to produce a realistic and reproducible benchmark environment for early fall-risk modelling.

### 3.7. Feature Simulation and Trajectory Design

Each time-series sequence contained seven core dynamic features selected on the basis of their empirical links to fall risk: heart rate, heart rate variability, respiratory rate, systolic blood pressure, gait step-time variability, postural sway, and centre of pressure.

Normal sequences were sampled from plausible geriatric ranges with mild variability and low-amplitude Gaussian noise. Slip sequences introduced brief perturbations in the middle of the time window, followed by recovery. Pre-fall sequences followed a two-phase trajectory consisting of gradual deviation during the first 40 s and more rapid deterioration during the final 20 s, reflecting progressive instability prior to the simulated fall anchor. Additive Gaussian noise was included to emulate sensor noise, motion artifacts, and day-to-day variability [18,30,31].

### 3.8. Synthetic Signal Parameterization

To improve transparency and reproducibility, the synthetic data generator was parameterized using physiologically plausible ranges derived from published geriatric biomechanics, cardiovascular monitoring studies, and public fall datasets. Baseline values and temporal dynamics were designed to approximate patterns observed in older adults during normal activities, transient perturbations, and progressive balance deterioration.

Heart rate (HR) values for normal sequences were sampled primarily within resting elderly ranges (approximately 60–90 beats/min), with mild stochastic variability introduced over time. Heart rate variability (HRV) was simulated using low-frequency fluctuations consistent with reduced autonomic adaptability commonly observed in frail older adults. Respiratory rate (RR) values were generated within typical resting ranges (approximately 12–20 breaths/min), while systolic blood pressure (SBP) values were simulated around normotensive elderly ranges with intermittent transient decreases during instability events to emulate orthostatic effects.

Biomechanical variables were parameterised using simplified representations of age-related gait and balance deterioration. Gait step-time variability (GaitVar) was modelled with relatively stable low variance during normal activities, transient spikes during slip events, and gradually increasing variance during pre-fall sequences. Postural sway and centre-of-pressure (CoP) trajectories were generated using bounded stochastic drift processes, with progressively increasing amplitude and directional instability during pre-fall windows.

Three temporal progression profiles were implemented:

- Normal ADL sequences: Signals fluctuated around stable baselines with low-amplitude Gaussian perturbations and no persistent directional drift.
- Slip or misstep sequences: A transient perturbation was introduced approximately mid-sequence (30–35 s), causing abrupt but recoverable deviations in selected physiological and biomechanical variables before returning toward baseline stability.
- Pre-fall sequences: A two-stage deterioration process was implemented. The first stage involved gradual increases in sway, CoP drift, and gait variability over approximately 40 s, followed by a second stage characterised by accelerated instability and amplified fluctuations approaching the simulated fall-onset anchor at the end of the sequence window.

To emulate real-world wearable acquisition conditions, additive Gaussian noise (10–15%) was applied independently to each signal channel. This was intended to approximate sensor noise, motion artefacts, and minor temporal inconsistencies commonly observed in wearable monitoring systems. While Gaussian perturbation provides a simplified approximation of wearable noise characteristics, we acknowledge that real-world artefacts may exhibit non-Gaussian behaviour, sensor drift, correlated channel noise, and intermittent signal dropout. These factors will be explored in future work using real-world datasets and more advanced simulation models.

In addition to dynamic sensor signals, static participant-level metadata (e.g., age, sex, medication exposure, fracture history, and bone mineral density) were sampled probabilistically from clinically plausible distributions informed by established geriatric risk assessment frameworks. These static covariates were integrated into each sequence to emulate inter-subject heterogeneity commonly observed in elderly populations.

### 3.9. Integration of Contextual Metadata

Each sample was enriched with static participant-level metadata sampled from clinically plausible ranges. These metadata included age, sex, height, weight, prior fracture, family history of hip fracture, smoking status, alcohol use, rheumatoid arthritis, glucocorticoid exposure, simulated femoral neck bone mineral density, and binary indicators for medication classes such as sedatives, antihypertensives, diuretics, and antidepressants. These contextual variables were intended to capture participant-specific risk factors that may improve model discrimination.

### 3.10. Label Noise Injection

To reflect ambiguity in real-world annotation, 5% of sequence labels were perturbed during dataset generation. Rather than modelling label uncertainty as entirely arbitrary class switching, perturbations were restricted primarily to behaviourally adjacent classes, especially slip and pre-fall states. This design reflects the fact that transient slips, recoveries, and early instability episodes may overlap in both kinematic expression and human annotation, whereas normal activity is less likely to be confused with imminent fall onset.

This label-noise mechanism was included to reduce overfitting to idealized class boundaries and to emulate uncertainty associated with near-fall and transition events in practice. The 5% noise level was selected as a conservative approximation of moderate annotation uncertainty. We did not model participant-specific, asymmetric, or temporally correlated mislabelling probabilities; these remain important extensions for future real-world validation.

### 3.11. Data Synchronization and Fusion

Dynamic sensor streams were assumed to be synchronized and aligned to a common temporal grid. Feature-level fusion was then used to combine the biomechanical, physiological, and contextual variables into a single multivariate representation for downstream learning. Standard preprocessing included feature normalization and fixed-length sequence representation.

### 3.12. Feature Extraction

The framework targeted two clinically relevant event types: imbalance points, representing early indicators of compromised balance from which recovery may still be possible, and fall points, representing moments immediately before a likely fall. The inclusion of imbalance points was intended to extend usable warning time beyond short pre-impact detection horizons and to support early intervention.

### 3.13. Sensor-Derived Feature Modelling and Evaluation

Eight baseline machine learning architectures were evaluated: Logistic Regression (LogReg), Random Forest, Extreme Gradient Boosting (XGBoost), Long Short-Term Memory (LSTM), Gated Recurrent Unit (GRU), Temporal Convolutional Network (TCN), Convolutional Neural Network–Long Short-Term Memory (CNN-LSTM), and Transformer.

Two additional augmented temporal models were also evaluated, namely a Temporal Convolutional Network with augmented input features and a lightweight Temporal Fusion Transformer (TFT-Lite) with augmented input features.

All models were trained using a 70/15/15 train/validation/test split performed subject-wise to reduce leakage. Hyperparameters were tuned using validation performance. Deep learning models were optimized using cross-entropy loss, while the Random Forest model used Gini impurity. Model performance was assessed using accuracy, macro F1-score, weighted F1-score, mean area under the receiver operating characteristic curve, and mean Average Precision. Table 1 summarises the machine learning metrics used in this study.

### 3.14. Time-Series Feature Augmentation

To enrich the representation of each dynamic channel without generating additional samples, we used signal-level feature augmentation. For each of the seven dynamic variables, we computed the first difference, a 5-s rolling mean, and a 5-s rolling standard deviation. A normalized time ramp was also appended to encode temporal position within the sequence window. These augmented descriptors were intended to expose short-horizon trend and volatility cues that may be relevant for early instability detection.

### 3.15. Model Training for Signal-Level Augmentation

The augmented models were trained using standard deep learning optimization procedures, including AdamW, learning-rate scheduling, and gradient clipping. Model selection was based on validation-set macro F1-score. The test set remained locked until the final evaluation. Rolling statistics and normalization were computed from the training partition only to avoid information leakage.

### 3.16. Real-Time Fall-Risk Prediction

The trained models were intended to operate on incoming data streams to produce continuously updated fall-risk estimates. These probabilistic outputs were then converted into alerts using thresholding and persistence logic.

### 3.17. Real-Time Alert Generation

Once risk scores exceeded the predefined threshold for the minimum persistence duration, the framework generated a pre-fall alert. This output was intended to support caregiver intervention during the critical warning period before a fall event.

## 4. Results

A simulation-based evaluation was conducted using the synthetic dataset of 1000 multimodal time-series sequences. Performance on the subject-wise test split is summarised in Table 2. Classical baselines achieved the strongest overall macro F1-scores, reflecting the influence of static covariates and summary-level separability within the synthetic generator. Among the temporal models, the Gated Recurrent Unit achieved the strongest macro F1-score, followed by the Temporal Convolutional Network and the Convolutional Neural Network–Long Short-Term Memory model. These results indicate that realistic subject-wise partitioning substantially reduces optimistic estimates observed under in-distribution splits.

### 4.1. Operational Early-Warning Performance

Because practical deployment requires converting model probabilities into alerts, we additionally evaluated a fixed alerting rule with a threshold of 0.70 and a persistence requirement of three consecutive samples. Results are shown in Table 3. Some models produced no alerts at this operating point, whereas others achieved higher pre-fall trigger rates at the cost of increased false alarms. These results show that classification performance alone does not directly translate into reliable operational behaviour.

### 4.2. Time-Series Feature Augmentation Results

Results for the augmented temporal models are shown in Table 4. Under the subject-wise evaluation protocol, augmentation altered model behaviour but did not produce near-perfect classification performance. The Temporal Convolutional Network with augmentation achieved a macro F1-score of 0.440, while the lightweight Temporal Fusion Transformer with augmentation achieved 0.390. Class-wise analysis showed that normal and pre-fall states were detected with moderate reliability, whereas slip remained the most difficult class.

### 4.3. Ablation and Interpretation of Baseline Performance

To better understand why several classical baselines outperformed temporal deep learning models under the subject-wise evaluation protocol, we conducted a supplementary ablation-style analysis examining the relative contribution of static participant metadata and dynamic sensor signals.

Three simplified input configurations were considered: (i) static covariates only (e.g., age, sex, medication exposure, fracture history, and bone mineral density), (ii) dynamic physiological and biomechanical signals only, and (iii) combined static and dynamic inputs. The analysis was intended to provide exploratory insight into the relative contribution of each modality rather than establish definitive clinical performance estimates.

As shown in Table 5, static metadata alone achieved higher performance than dynamic signals alone (Macro-F1 0.615 vs. 0.450), suggesting that geriatric risk factors contributed substantially to class separability in the synthetic benchmark. However, the combined input configuration achieved the strongest overall performance (accuracy 0.764, Macro-F1 0.698), indicating that dynamic signals provided complementary predictive information beyond static covariates alone.

These findings help explain why some classical models performed comparatively well under subject-wise splits. Because the synthetic generator incorporated clinically informed covariate distributions together with gradual temporal deterioration patterns, part of the separability could be captured through summary-level statistical relationships in addition to temporal dynamics. Future work using real-world longitudinal wearable datasets will be necessary to determine the extent to which advanced temporal architectures outperform classical baselines under clinically realistic conditions.

## 5. Discussion

This study presents a multimodal AI-driven framework for early fall-risk prediction and highlights the importance of an evaluation protocol when interpreting model performance. Under subject-wise splits, classical baselines leveraging static covariates achieved stronger overall discrimination than the temporal models, while the temporal models showed more modest but still informative performance. These findings suggest that apparent gains observed under in-distribution settings may not translate directly to more realistic deployment scenarios.

A key observation is that no single architecture uniformly dominated across all metrics. Instead, the temporal models exhibited distinct trade-offs between sensitivity, false-alarm rate, and lead time under the fixed alerting rule. This finding reinforces the need to evaluate fall prediction systems not only by aggregate classification scores but also by operational behaviour under realistic alert thresholds.

From a practical perspective, even a warning interval of tens of seconds may support preventative action; however, clinically useful lead time depends on probability calibration and threshold selection. Fixed-threshold analyses can yield either missed alerts or elevated false-alarm rates, which may undermine trust in a deployed system. Consequently, clinically meaningful warning performance should be treated as a deployment objective that requires explicit calibration and validation rather than as a direct consequence of classifier output alone.

The results also show that feature augmentation can alter model behaviour in useful ways, although it does not eliminate the challenges posed by subject-wise generalization and class overlap. In particular, slip events remained difficult to classify, likely because their transient signatures overlap with both normal variability and pre-fall instability. Future work should include ablation studies to isolate the contribution of individual augmentation components and assess robustness under alternative generators and real-world datasets.

### 5.1. Wearable Sensing Compared with WiFi CSI-Based Approaches

Recent work has explored WiFi Channel State Information (CSI) as a device-free and privacy-preserving alternative for human activity recognition and pose estimation. For example, CSI-based methods have been combined with skeleton-based modelling, YOLOv8, and MediaPipe frameworks for simultaneous activity recognition and pose estimation, and other recent systems have used WiFi CSI for low-cost indoor activity recognition without requiring participants to wear sensors [32,33]. These approaches are highly relevant to fall prediction because they avoid user adherence issues associated with wearables and may be suitable for unobtrusive monitoring in instrumented indoor environments. Recent transformer-based camera and human pose-estimation systems have also demonstrated the feasibility of lightweight, privacy-preserving fall monitoring at the edge, including implementations designed for low-power devices such as Raspberry Pi platforms [34].

However, WiFi CSI and wearable sensing address partially different deployment constraints. CSI-based systems can support device-free monitoring and may reduce concerns related to cameras or body-worn compliance, but they may be sensitive to environmental layout, multipath effects, room configuration, and changes in occupancy. In contrast, wearable sensors can capture subject-specific physiological and biomechanical signals such as heart rate, blood pressure, gait variability, and postural sway, which are central to the proposed FPS framework. Thus, rather than viewing CSI-based and wearable approaches as competing paradigms, future work could extend the proposed benchmarking framework to evaluate hybrid systems that combine ambient CSI-derived movement cues with wearable physiological and biomechanical signals.

### 5.2. Deployment Considerations for Wearable Early-Warning Systems

Practical deployment of wearable fall-prediction systems requires attention to sensing reliability, energy consumption, computational latency, and alert burden. The proposed framework uses 60-s windows sampled at 5 Hz, corresponding to 300 time steps per sequence, which is compatible with low-rate wearable monitoring and substantially less demanding than high-frequency inertial or video-based systems. However, real-time operation would require continuous window updating, efficient feature extraction, and careful management of battery consumption on body-worn devices.

In deployment, model inference could be performed either on-device, on a paired smartphone, or on an edge/cloud service, depending on privacy, latency, and power constraints. Classical models and compact temporal architectures, such as TCN-based models, are likely more suitable for edge deployment than larger attention-based models because they require fewer sequential operations and can process time windows efficiently. Nevertheless, formal benchmarking of inference time, memory use, and battery impact on embedded hardware was outside the scope of the present simulation study.

User safety also depends on the alerting policy. Excessive false alarms may lead to alert fatigue among caregivers or older adults, whereas overly conservative thresholds may fail to provide actionable warnings. Therefore, deployment would require calibration of thresholds and persistence rules to meet context-specific constraints, such as acceptable false alarms per day, minimum desired lead time, and available caregiver response pathways. Future work will evaluate the FPS framework on embedded platforms and in realistic monitoring workflows to quantify inference latency, energy use, and user acceptance.

Several limitations should be acknowledged. First, all experiments were conducted using a synthetic data generator informed by empirical priors rather than real-world prospective data. Second, although the benchmark incorporates multiple modalities and contextual variables, it remains a simulation environment and cannot fully reproduce deployment conditions, sensor artifacts, or annotation uncertainty in clinical settings. Third, some of the strongest performance in the synthetic benchmark appears to derive from static covariates and summary-level separability, which may not persist in real-world populations. Although the present benchmark is synthetic, it is designed to support future evaluation of multimodal wearable sensing pipelines under controlled conditions before deployment on real sensor platforms.

Despite these limitations, the proposed framework provides a reproducible foundation for comparing time-series AI architectures under controlled conditions. This benchmark can support future work on calibration, edge deployment, multimodal sensor fusion, and prospective validation in older adult cohorts.

## 6. Conclusions

This study introduced an AI-powered Fall Prediction System framework using multi-sensor fusion and time-series machine learning models. Results from synthetic data showed that model performance was highly sensitive to evaluation protocol and alerting rules, with temporal and augmented models exhibiting distinct trade-offs between sensitivity and false-alarm rate under subject-wise evaluation. In particular, converting model outputs into reliable alerts requires explicit threshold selection and calibration.

These findings suggest that temporal and feature-augmented models can provide a useful foundation for early-warning systems when paired with careful calibration and validation, complementing strong baseline performance from classical models. Beyond technical performance, the proposed framework offers a reproducible basis for future work on fall-risk prediction in digital health settings. With further validation in real-world clinical cohorts, these models may support proactive intervention strategies aimed at reducing fall-related injuries and supporting ageing in place.

## Figures and Tables

**Table 1:** Machine learning metrics used in this study.

Metric	Definition	Description
Accuracy	Proportion of correct predictions out of all predictions.	Good when classes are balanced. Can be misleading if one class dominates (e.g., 95% accuracy by always predicting the majority class).
F1-score (macro)	Average of F1-scores across all classes, treating each class equally (unweighted).	Good for imbalanced datasets, since rare classes are weighted the same as common ones.
F1-score (weighted)	Weighted average of F1-scores, where weights are proportional to class frequency.	Good for assessing overall performance while still accounting for class imbalance and reflecting dataset distribution.
Mean AUROC	Area under the receiver operating characteristic curve, averaged across classes. Measures the ability to separate positive versus negative cases.	Useful for probabilistic models and for assessing how well the model ranks positive examples higher than negative examples.
Mean AP	Mean Average Precision (area under the precision–recall curve). Focuses on precision versus recall.	Very useful for imbalanced datasets where AUROC may appear overly optimistic.

**Table 2:** Model performance summary on the subject-wise test split.

#	Model	Accuracy	F1-Score (Macro)	F1-Score (Weighted)	Mean AUROC	Mean AP
1	LogReg	0.7557	0.6814	0.7347	0.8523	0.7734
2	RandomForest	0.7557	0.6749	0.7344	0.8099	0.7404
3	XGBoost	0.7634	0.6809	0.7409	0.8237	0.7532
4	LSTM	0.3511	0.3650	0.3209	0.6790	0.5500
5	GRU	0.4656	0.4650	0.4749	0.6629	0.5302
6	TCN	0.5344	0.3954	0.4990	0.6337	0.5296
7	CNN-LSTM	0.5267	0.3927	0.4786	0.6305	0.4613
8	Transformer	0.5573	0.3626	0.4932	0.6168	0.4651

Note: Classical baselines leverage static covariates and summary-level separability in the synthetic generator; subject-wise splits reduce optimistic estimates observed under in-distribution splits.

**Table 3:** Operational early-warning performance on the subject-wise test split using a fixed alerting rule (threshold = 0.70; persistence = 3 consecutive steps).

Model	Pre-Fall Trigger Rate	Non-Fall False-Alarm Rate	Lead Time (Median, s)	Lead Time (p25, s)	Lead Time (p75, s)
GRU	0.7391	0.2870	11.8	8.2	29.8
CNN-LSTM	0.6087	0.2685	18.0	11.5	36.4
TFT-Lite	0.2609	0.1944	35.0	26.9	48.5
TCN + Aug (causal)	0.2174	0.0278	26.6	26.2	29.4
TCN (causal)	0.1739	0.1019	24.6	23.1	26.6
LSTM	0.0000	0.0000	NA	NA	NA
Transformer	0.0000	0.0000	NA	NA	NA
TFT-Lite + Aug	0.0000	0.0000	NA	NA	NA

Notes: (1) Pre-fall trigger rate is the fraction of true pre-fall sequences that triggered an alert; non-fall false-alarm rate is the fraction of non-fall sequences (normal + slip) that triggered. Lead time is reported only for triggered pre-fall sequences. (2) “NA” means no pre-fall triggers occurred at this operating point, so lead time is undefined.

**Table 4:** Class-wise test performance of TCN + Augmentation and TFT-Lite + Augmentation on the subject-wise test split using augmented input representations.

Model	Class	Precision	Recall	F1-Score	Accuracy/Macro-F1
TCN + Aug	0 (Normal)	0.622	0.718	0.667	Acc = 0.542, Macro-F1 = 0.440
TCN + Aug	1 (Slip)	0.211	0.133	0.163	
TCN + Aug	2 (Pre-fall)	0.500	0.478	0.489	
TFT-Lite + Aug	0 (Normal)	0.597	0.551	0.573	Acc = 0.458, Macro-F1 = 0.390
TFT-Lite + Aug	1 (Slip)	0.167	0.133	0.148	
TFT-Lite + Aug	2 (Pre-fall)	0.371	0.565	0.448	

**Table 5:** Ablation comparison of static and dynamic feature groups on the subject-wise test split.

Input Configuration	Accuracy	Macro-F1	Interpretation
Static metadata only	0.679	0.615	Static covariates show moderate class separability.
Dynamic signals only	0.531	0.450	Dynamic signals show weaker but detectable temporal information.
Combined static and dynamic inputs	0.764	0.698	Combined inputs perform best, indicating complementary value.

## Data Availability

The synthetic datasets and machine learning code used in this study are available from the corresponding author on reasonable request.
